# Oxidative lesions and post‐treatment viability attenuation of *listeria monocytogenes* triggered by atmospheric non‐thermal plasma

**DOI:** 10.1111/jam.15688

**Published:** 2022-08-03

**Authors:** Yuanyuan Pan, Jun‐Hu Cheng, Da‐Wen Sun

**Affiliations:** ^1^ School of Food Science and Engineering South China University of Technology Guangzhou China; ^2^ Academy of Contemporary Food Engineering South China University of Technology, Guangzhou Higher Education Mega Center Guangzhou China; ^3^ Engineering and Technological Research Centre of Guangdong Province on Intelligent Sensing and Process Control of Cold Chain Foods, & Guangdong Province Engineering Laboratory for Intelligent Cold Chain Logistics Equipment for Agricultural Products Guangzhou Higher Education Mega Centre Guangzhou China; ^4^ Food Refrigeration and Computerized Food Technology (FRCFT), Agriculture and Food Science Centre University College Dublin, National University of Ireland Dublin Ireland

**Keywords:** DBD plasma, DNA lesions, gene expression, *L. Monocytogenes*, virulence

## Abstract

**Aims:**

The aim of the current study was to investigate the effect of plasma‐mediated oxidative stress on the post‐treatment viability of *Listeria monocytogenes* at the physiological and molecular levels.

**Methods and Results:**

10^7^ CFU/ml *L. monocytogenes* in 10 ml phosphate‐buffered saline (PBS) was treated with atmospheric non‐thermal plasma for 0, 30, 60, 90 and 120 s respectively. Optical diagnostics using optical emission spectroscopy (OES) confirmed that dielectric barrier discharge (DBD) plasma was a significant source of ample exogenous reactive oxygen and nitrogen species (RONS). The development of extracellular main long‐lived species was associated with plasma exposure time, accompanied by a massive accumulation of intracellular ROS in *L. monocytogenes* (*p* < 0.01). With the exception of virulence genes (*hly*), most oxidation resistance genes (e.g. *sigB*, *perR*, *lmo2344*, *lmo2770* and *trxA*) and DNA repair gene (*recA*) were upregulated significantly (*p* < 0.05). A visible fragmentation in genomic DNA and a decline in the secretion of extracellular proteins and haemolytic activity (*p* < 0.01) were noticed. The quantitate oxygen consumption rates (OCRs) and extracellular acidification rates (ECARs) confirmed the viability attenuation from the aspect of energy metabolism. Survival assay in a real food system (raw milk) further suggested not only the viability attenuation, but also the resuscitation potential and safety risk of mild plasma‐treated cells during post‐treatment storage.

**Conclusion:**

DBD plasma had the potential to inactivate and attenuate the virulence of *L. monocytogenes*, and it was recommended that plasma exposure time longer than 120 s was more suitable for attenuating viability and avoiding the recovery possibility of *L. monocytogenes* in raw milk within 7 days.

**Significance and Impact of the Study:**

The current results presented a strategy to inactivate and attenuate the viability of *L. monocytogenes*, which could serve as a theoretical basis for better application of non‐thermal plasma in food in an effort to effectively combat foodborne pathogens.

## INTRODUCTION

*Listeria monocytogenes*, as a foodborne pathogen, has a profound potential to survive and grow over a wide range of temperatures between 1 and 45°C and is potentially lethal in immunocompromised individuals (Radoshevich & Cossart, 2018). During food processing and storage, *L. monocytogenes* is commonly exposed to oxidative stress caused by processing techniques and/or disinfectants (Bansal et al., 2018). The ability of *L. monocytogenes* to adapt to a wide range of oxidative stress has also been extensively studied and reviewed in an attempt to identify and understand the factors behind this efficient adaptation (Abeysundara et al., 2016). However, its growth in low temperatures and high adaptation ability to physicochemical interventions make it more difficult to be controlled in ready‐to‐eat foods (Azizoglu & Kathariou, 2010).

Nowadays, xcold plasma is increasingly explored as an effective non‐thermal technique for the inactivation of micro‐organisms in a wide range of food sectors (Ali et al., 2021a, Ali et al., 2021b; Esua et al., 2021a, 2021b; Bourke et al., 2018; Pan et al., 2021). During plasma discharge in air or gas–liquid interface, the agents generated include reactive oxygen species (ROS) (e.g. hydrogen peroxide H_2_O_2_, ozone O_3,_ singlet oxygen ^1^O_2_, atomic oxygen O, superoxide anion O_2_‐, peroxide O_2_^−2^, hydroxyl radicals OH• and hydroxyl ions OH‐), reactive nitrogen species (RNS) (e.g. atomic nitrogen N, excited nitrogen N_2_[A] and nitric oxide NO•), ultraviolet radiation, energetic ions and charged particles, which have been demonstrated to be effective in inducing oxidative stress and inactivating micro‐organisms (Ali et al., 2022; Ekezie et al., 2019a, 2019b; Misra et al., 2011; Pan et al., 2019a; Yost & Joshi, 2015). The adverse environmental conditions (e.g. oxidative stress) derived by these reactive species can easily induce SOS (derived from ‘Save Our Souls’) response of micro‐organisms (Liu et al., 2018; van der Veen et al., 2010; Wu et al., 2022).

Micro‐organisms can develop antioxidant systems to avoid or reduce oxidative damage. These defence systems include (i) genes encoding enzymes that scavenge reactive oxygen, such as superoxide dismutase (*sod*), catalase (*kat*) and Ferritin‐like protein (*fri*); (ii) stress regulators that control some antioxidant genes expression, such as *perR* and *sigB*; and (iii) DNA repair genes that restore damaged DNA, such as *recA* (Suo et al., 2014). For example, Yost and Joshi (2015) found that the oxidative response of *Escherichia coli* to plasma‐activated phosphate‐buffered saline (PBS) was predominantly regulated by OxyR and SoxyRS regulons, accompanied by significant overexpression of *kat*, *sod* and *recA* genes. In addition, nuclear magnetic resonance (NMR) was utilized by Liu et al. (2018) to determine the modifications in metabolite profiling of *Listeria innocua* after treatment by electrolysed water and the results indicated that electrolysed water perturbation inhibited a series of biochemical processes (e.g. peptidoglycan synthesis, nucleotides biosynthesis and amino acid metabolism), which also activated some oxidative stress defence systems such as glutamate decarboxylase system. However, the mode of action and the resulting oxidative stress response of *L. monocytogenes* to plasma exposure at the gene transcription level have not yet been fully investigated to date.

Therefore, the objective of the current study was to elucidate the plasma‐mediated variations in the post‐treatment viability of *L. monocytogenes* and understand its oxidative stress response mechanism to cold plasma. In this study, optical emission spectroscopy (OES) was employed to characterize the reactive species generated in the plasma gas phase. Main extracellular long‐lived species and intracellular ROS levels were also determined to evaluate the extracellular and intracellular oxidative stress levels that *L. monocytogenes* suffered during plasma treatment. The transcriptomic analysis was applied using real‐time quantitative reverse transcription‐polymerase chain reaction (qRT‐PCR) to determine the transcription regulation levels of stress or virulence genes following plasma exposure. Agarose gel electrophoresis and enzyme‐linked immunosorbent assay (ELISA) were adopted to confirm the DNA oxidative lesions after plasma treatment at the molecular level. SDS‐PAGE (sodium dodecyl sulphate polyacrylamide gel electrophoresis) and haemolysis assays were adopted to show the secretion of extracellular proteins and haemolytic activity in *L. monocytogenes* after plasma treatment, respectively. In addition, the quantitate oxygen consumption rates (OCRs) and extracellular acidification rates (ECARs) were adopted to confirm the viability attenuation from the aspect of energy metabolism. Finally, the survival and growth assay in raw milk were adopted to determine the post‐treatment viability and recovery possibility as well as the potential risk in a real food system during post‐treatment storage.

## MATERIALS AND METHODS

### Strain and growth conditions

Gram‐positive *L. monocytogenes* ATCC 19115 (G^+^), as the standard reference microbial strain, was purchased from Guangdong Microbial Culture Center (GDMCC) (Guangzhou, China) and stored at 4–8°C. A standard protocol described by GDMCC was adopted to activate the strain. A loopful of the isolated colony was then transferred to sterile tryptic soy broth with 0.6% of yeast extract (TSB‐YE) and incubated at 37°C until the desired exponential phase was achieved. After incubation, the cells were harvested, washed and resuspended in the sterile cold (4°C) PBS (0.1 mol/L, pH 6.0) with a final concentration of approximately 10^7^ CFU/ml.

### DBD plasma system and treatments

The configuration of the DBD plasma system (CTP‐2000 K, Nanjing Suman Electronics Co., Ltd.) utilized in the current study was installed in a sterile laminar flow cabinet, and the details were described in our previous study (Pan et al., 2020), mainly the system consisted of a high‐frequency alternating current power source (CTP‐2000 K) and a DBD plasma reactor (DBD‐50) containing two steel electrodes (Φ50 mm) and an upper quartz plate (Φ102 × 1 mm). For plasma treatment experiments, 10 ml bacterial suspension was transferred to a sterilized petri dish (Ф 60 mm) and an operating input voltage of 50 V, an input power of 1000 W, a frequency of 10 kHz and a discharge gap of 5 mm between the quartz plate and sample surface were employed in the experiment. In addition, plasma exposure periods were set as 0, 30, 60, 90 and 120 s, respectively.

### Optical diagnostics of air plasma

OES was employed for optical diagnostics of the reactive gas species generated by the DBD plasma in the gas phase. The emission spectra were measured using a computer‐controlled HR2000+ spectrometer (Ocean Optics Inc.) with a 0.66 nm optical resolution and 1000 mm optical fibres. In addition, the fibres had a numerical aperture of 0.22 and were optimized for use in the ultraviolet and visible portions of the spectra with a wavelength range between 200 and 1100 nm. The data were analysed using OceanView Optics software (Ocean Optics Inc.). The peaks were identified by comparing with the NIST Atomic Spectra Database (Xu et al., 2017).

### Physicochemical properties and diagnosis of intracellular ROS levels

A pH meter (SP‐2300, Suntex Instruments Co., Ltd.) and an oxidation–reduction potential (ORP) probe with an Ag/AgCl electrode (Mettler Toledo Instruments Co. Ltd.) were utilized to detect the pH and ORP values of untreated and treated bacterial suspensions at 25°C, respectively. The concentrations of H_2_O_2_ in suspensions were quantified by a hydrogen peroxide assay kit (Sangon Biotech Co., Ltd.), while the concentration of nitrate (NO_3_^−^) and nitrite (NO_2_^−^) in suspensions were measured according to the method described by Shen et al. (2016).

Flow cytometry (FCM) in tandem with fluorescent techniques was adopted to determine the levels of intracellular oxidative stress (ROS) suffered by *L. monocytogenes* after plasma treatment. As usual, 2′,7′‐dichloro‐dihydro‐fluorescein diacetate (DCFH‐DA) (Sigma‐Aldrich Trading Co., Ltd.) was chosen as the cellular assay probe (Pan et al., 2019b).

### RNA extraction and real‐time qRT‐PCR analysis

The extraction of total RNA was carried out by following the manufacturer's instructions of a UNIQ‐10 column Trizol total RNA extraction kit (Sangon Biotech Co., Ltd.) and an iScript cDNA synthesis kit (Bio‐Rad Laboratories, Inc.), respectively. Before RNA extraction, 20 mg/ml lysozyme solution (Tiangen Biotech Co., Ltd.) was added to pellets and incubated at 37°C for 40 min to lyse the cells. The purity and concentration of the extracted RNA were checked using a microspectrophotometer (SMA4000, Merinton Instrument, Inc.), and the integrity of extracted RNA was measured by 1.5% agarose gel electrophoresis.

For the first‐strand cDNA synthesis, 800 ng of total RNA was used for reverse transcription according to the instructions of the iScript cDNA synthesis kit (Bio‐Rad Laboratories, Inc.). Real‐time qRT‐PCR was performed using a StepOnePlus Real‐Time PCR System (ABI) to evaluate the transcriptional levels of stress/virulence‐related genes. The specific primers of genes and associated functional descriptions were designed and synthesized by Sangon Biotech Co. Ltd., which are listed in Table 1. The relative expressions of these stress/virulence‐related genes in *L. monocytogenes* were calculated using the 2^−△△Ct^ method (Li et al., 2019).

**TABLE 1:** The specific primers of genes and associated functional descriptions used in this study

Gene	Sequence (5′ to 3′)	Product length (bp)	Functional description	References
*16SrRNA*	F: CCTACGGGAGGCAGCAG R: ATTACCGCGGCTGCTGG	169	Housekeeping gene	Huang et al. (2018)
*sigB*	F: ATTTGGATTGCCGCTTACC R: TCGGGCGATGGACTCTACT	93	General stress transcription factor	Huang et al. (2018)
*PerR*	F: CGTTTTCCGTGATGCTGGT R: TGTGCTGCGAAATGTTCTACTT	165	Oxidative stress transcriptional regulator	Huang et al. (2018)
*kat*	F: TTGACCGCAACCCAGATAA R: GGCCCTACACGATGTCTTTG	151	Encoding catalase	Huang et al. (2018)
*sod*	F: CGTTCTTGGCTTAGATGTTTGG R: CGTTTGTTAGCTTCATCCCAGT	114	Encoding superoxide dismutases	Huang et al. (2018)
*prxs*	F: TTGAGGAAGAAGGCGTAGCA R: GGCATAGTCCACCAGTTTGAAG	150	Encoding peroxides enzymes	Dons et al. (2014)
*lmo2344*	F: ACCGACCATGATTATTTGCG R: GATTTCTGTAACAGCTTGGTAGGT	114	Encoding glutaredoxin to regulate cellular redox levels	Srinivas and Gopal (2014)
*lmo2770*	F: CTGGTTGCTTATTTATTTAACTGGC R: GCTACTACCGTCTGGAAGGGAT	94	Encoding glutathione synthetase	Srinivas and Gopal (2014)
*trxA*	F: TTGAACAAGAAACTAGCGAAGG R: GGGTTTTCATCTACGTCCATTT	151	Encoding thioredoxin reductase and thioredoxin	Dons et al. (2014)
*recA*	F: GCTTTAGGAGTTGGCGGATAC R: GCTTGTTCTCCTGTATCTGGTTG	227	DNA repair gene	Huang et al. (2018)
*prfA*	F: TGCCAGAAGCGGAAGAAGT R: TCGAAACGACAATTCCGGT	150	Central virulence regulator of virulence gene transcription	Huang et al. (2018)
*hly*	F: TCAGCATTGATTTGCCAGGTAT R: TTCACTGTAAGCCATTTCGTCA	182	Encoding Listeriolysin O	Pushkareva and Ermolaeva (2010)

### DNA lesions

The electrophoresis technique was applied using a gel set (DYCP‐31DN, Beijing Liuyi Instrument Factory, Beijing, China) to investigate the impact of plasma treatment on genomic DNA integrity (Yost & Joshi, 2015). First, the DNA of plasma‐treated or untreated samples was isolated and purified according to the protocol of a bacterial DNA isolation kit (Tiangen Biotech Co., Ltd.), and 0.8% agarose gel electrophoresis was then used to demonstrate the physical disintegration of DNA.

In addition, 8‐hydroxydeoxyguanosine (8‐OHdG), which is a specific biochemical marker of oxidatively damaged DNA, was used to identify the extent of oxidative damage to DNA with an oxidative DNA damage ELISA kit (Shanghai Yuanmu Biological Technology Co.), which could provide rapid detection and quantification of intracellular 8‐OHdG after plasma exposure.

### Secretion of extracellular proteins

After plasma treatment for different durations, the untreated and treated samples were inoculated in Luria‐Bertani (LB) broth at 37°C until the OD_600_ was 0.5–0.6. The harvested bacterial culture was then filtered through a sterile syringe filter (cellulose acetate, 0.22 μm/13 mm, Sangon Biotech Co., Ltd.), which was used for subsequent assays.

#### SDS‐PAGE assay

After filtration, 6 ml of the supernatant was mixed with 100 μl of 2% sodium deoxycholate (Sangon Biotech Co., Ltd.) at room temperature of 25°C for 10 min, and then extracellular proteins were precipitated on ice using 2 ml of 10% trichloroacetic acid (Sigma‐Aldrich Trading Co., Ltd.) for 4–5 h. Next, after centrifugation at 20,800 g for 40 min at 4°C, the supernatant was removed and the pellet was resuspended twice in 1 ml of 80% ice‐cold acetone. Finally, the dried pellet was resuspended in 40 μl 0.01 mol/L PBS and stored at −20°C for SDS‐PAGE assay.

An amount of 9 μl of the above protein solution was mixed with 7.5 μl NuPAGE 4 × loading buffer and 13.5 μl deionized water and boiled at 100°C for 10 min. After a short vortex, SDS‐PAGE separation was performed. The electrophoresis was conducted with a Novex XCell SureLock Mini‐Cell (Invitrogen™, Thermo Fisher Scientific Inc.) using NuPAGE™ 4–12% Bis‐Tris precast gels and 1× NuPAGE™ MES‐SDS running buffer (Invitrogen™, Thermo Fisher Scientific Inc.), by running at 110 mA for 40–50 min. After electrophoresis, the gels were stained with Coomassie Brilliant Blue R‐250 (Invitrogen™, Thermo Fisher Scientific Inc.) and decolourized with deionized water overnight. Finally, an Amersham Imager 600 (GE Healthcare Bio‐Sciences AB) was used to image the gels.

#### Haemolysis assay

For haemolysis assay, 1 ml defibrinated sheep red blood cells (SRBC) (Zhengzhou Yikang Bio‐engineering Co., Ltd.) was suspended in 9 ml 0.01 mol/L PBS (pH 7.4) and centrifuged at 900 *g* for 15 min at 4°C. The precipitated pellet of SRBC was resuspended and washed in PBS until the supernatant was visibly clear. An amount of 1 ml sterile filtered supernatant was mixed with 2 mM dithiothreitol (DTT) (Sangon Biotech Co., Ltd.) to fully activate Listeriolysin O (LLO). Subsequently, the mixture and 10% SRBC solution were added to a 96‐well fluorescence microplate well at a volume ratio of 1: 9 and incubated at 37°C for 30 min. Thereafter, 0.1% sodium dodecyl sulphate (SDS) (Sangon Biotech Co., Ltd.) and LB broth were added to 10% SRBC solution as a positive control and a negative control, respectively. Finally, a centrifuge (Allegra X‐15R, Beckman Coulter Inc.) equipped with a 96‐well plate carrier adaptor was used to centrifuge the mixture at 3250 *g* for 40 min at 4°C. After centrifugation, the supernatant was transferred into a new 96‐well plate and the absorbance was measured on a SpectraMax® i3 plate reader (Molecular Devices) at 541 nm.

### Bacterial respiration

Bacterial respiration was determined according to the method described by Dwyer et al. (2014) with slight modifications. OCRs and ECARs were quantitated using the XFe96 Extracellular Flux Analyser (Seahorse Bioscience, Agilent Technologies) and the basal energy metabolism of cells was further assessed by analysing OCR/ECAR ratios (Smith et al., 2020).

After plasma treatment, cultures were washed and diluted to OD_600_ of 0.02 in TSB‐YE and 90 μl of diluted cells were added to XF cell culture microplates precoated with poly‐d‐lysine (PDL). Cells were centrifuged for 30 min at 250 *g* in a Heraeus Megafuge 40R centrifuge (ThermoFisher Scientific) to attach them to the pre‐coated plates, followed by raising the well volume to 180 μl in each well by gentle addition of 90 μl TSB‐YE. To assure uniform cellular seeding, initial OCR was measured for two cycles (7 min).

### Survival and growth of *L. monocytogenes* in raw milk

An amount of 1 ml plasma untreated or treated *L. monocytogenes* suspension was inoculated in 9 ml raw milk (Guangdong Yantang Dairy Industry Milk Co. Ltd.) and stored at 4°C for 0 and 7 days for microbiological enumeration. All plates using tryptic soy agar were incubated at 37°C from 24 to 48 h, and the results were expressed in log CFU/ml.

### Statistical analysis

OriginLab 8.0 software (OriginLab Inc.) was adopted to perform statistical analysis (Tian et al., 2020a, 2020b). The differences among plasma untreated and treated samples were further determined by the least significant difference test (LSD) with SPSS software (version 20.0) (IBM Analytics) at *p* < 0.05 level (Zhang et al., 2018). Each experiment was repeated in triplicate or quadruplicate and averaged data were reported.

## RESULTS

### Diagnosis of reactive gas species

**FIGURE 1:**
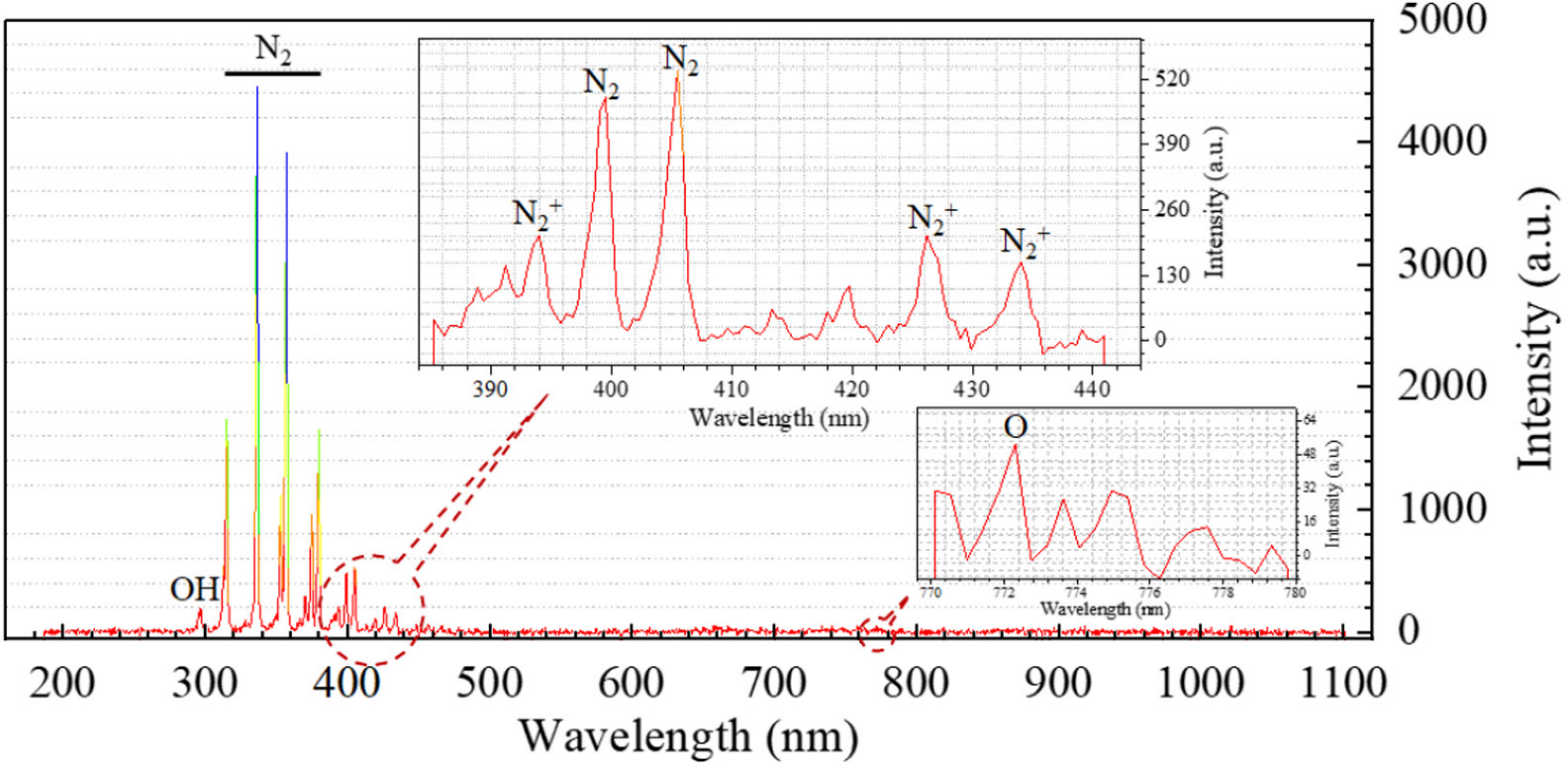
The main peaks in OES corresponding to the emissions of excited species generated by DBD plasma in air at 50 V over the range of 200–1100 nm

Figure 1 shows the main peaks of OES corresponding to the emissions of excited species generated by DBD plasma in the gas phase. The bulk of the observed emission was in the near‐UV region (300–400 nm), corresponding to the vibrational structure of electronic transitions of common diatomic molecules (e.g. OH, N_2_). In detail, the peak around 296.92 nm was attributed to the optical transition of OH (Connolly et al., 2013). Similar to the results of Walsh et al. (2010) and Xu et al. (2017), bands located between 300 and 383 nm as well as around 399.52 and 405.51 nm were assigned to the second positive system N_2_(C‐B), which corresponded to the transition between C^3^∏_u_ and B^2^Π_g_ electronic states, whereas the bands around 393.98, 426.22 and 434.03 nm were mainly due to the first negative system N_2_^+^(B‐X), which corresponded to the transition between B^2^∑_u_^+^ and X^2^∑_g_^+^ electronic states. Emissions from the O atom were only observed at 772.3 nm with low intensity.

### Physicochemical properties and intracellular oxidative stress levels

**FIGURE 2:**
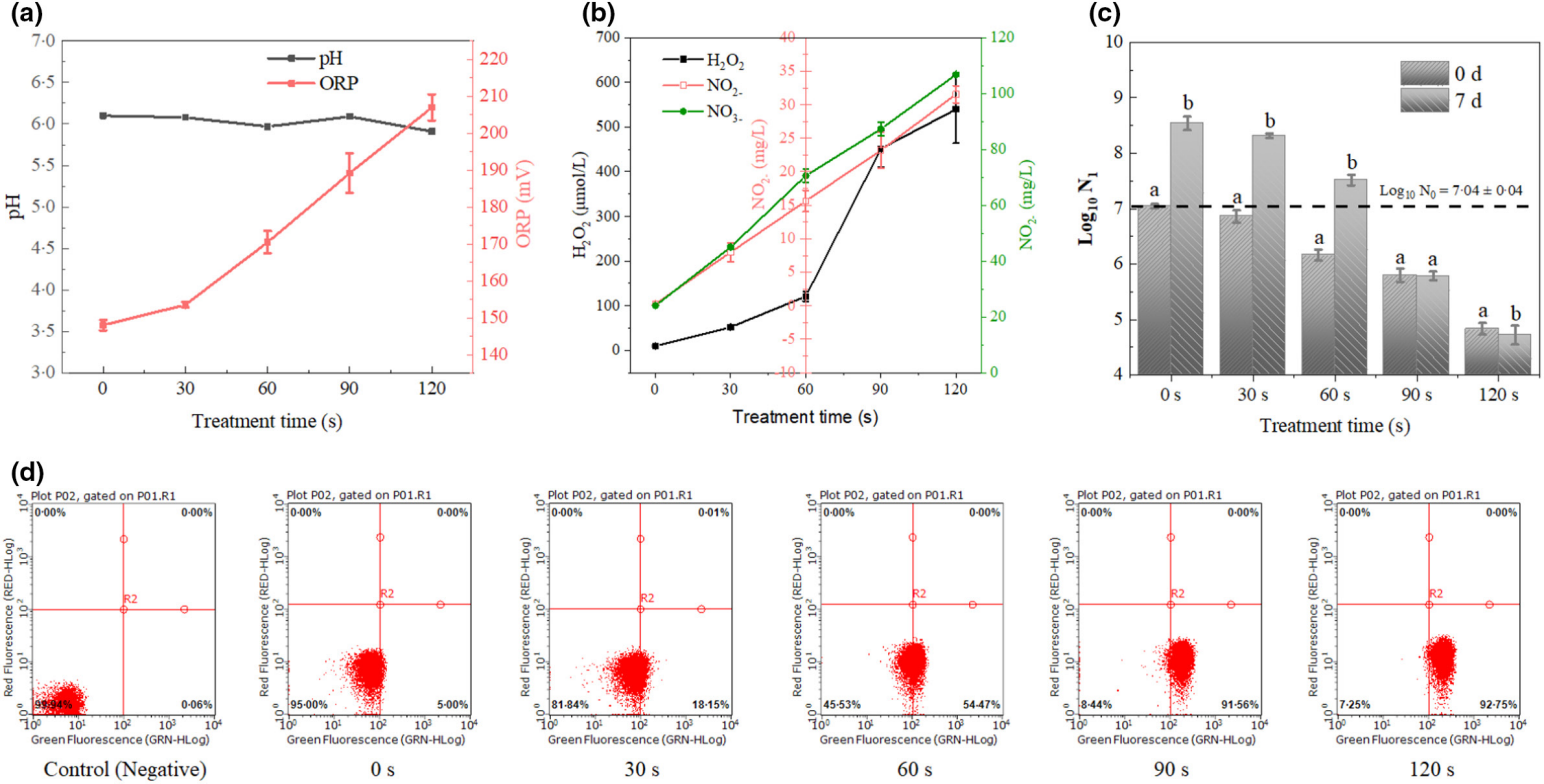
The development of (a) pH/ORP and (b) H_2_O_2_/NO_3_^−^/NO_2_^−^ in bacterial suspension subjected to DBD plasma treatment for different times; (c) post‐treatment viability of *L. monocytogenes* in raw milk at 4°C after plasma treated for different times; (d) percentage of DCF‐stained *L. monocytogenes* cells measured by FCM after exposure to plasma treatment. The values given in the figure represent the mean ± SD of triplicate samples. Values in the same figure with different letters were significantly different (*p* < 0.05)

The development of physicochemical properties (pH/ORP, H_2_O_2_, NO_3_^−^ and NO_2_^−^) of plasma‐treated bacterial suspensions for different durations is depicted in Figure 2a,b, which indicated the extracellular oxidative environment that *L. monocytogenes* was exposed to. Obvious changes were not observed in pH values, which were stabilized at around 6.0 even when the plasma exposure time was extended to 120 s. Thus, the inactivation of *L. monocytogenes* in the current study might not be attributed to the changes in acidity effects of the liquid media. As displayed in Figure 2a, a significant elevation was observed in ORP values as plasma exposure time progressed (*p* < 0.01), implying an accumulation of reactive chemical species in the liquid phase. As shown in Figure 2b, abundant H_2_O_2_ accumulated in bacterial suspension during plasma treatment for 120 s (*p* < 0.01), which was in accordance with previous studies (Shen et al., 2016). Figure 2b also indicated that the concentrations of NO_3_^−^ and NO_2_^−^ exhibited time‐dependent behaviour, which gradually accumulated as plasma treatment time progressed.

In addition, FCM in tandem with fluorescent techniques was adopted to determine the intracellular ROS level of *L. monocytogenes* after each treatment time, which directly represented the intensity of the intracellular oxidative stress, to which cells were subjected. Compared with the untreated samples, a progressive and significant elevation of intracellular ROS level was observed in *L. monocytogenes* cells (*p* < 0.01) (Figure 2d). In detail, plasma treatment for 90 s was enough to elevate the percentage of highly fluorescent 2′,7′‐dichlorodihydrofluorescein (DCF) stained cells for up to 90%, implying that plasma treatment for only 90 s triggered almost 16.75‐fold over‐oxidative stress than the oxidative stress induced by normal aerobic metabolism (5.43% ± 0.62). With increasing plasma exposure time, the percentage of DCF stained cells was further elevated to 94.31% ± 1.49 after 120 s.

### Expression verification of stress/virulence‐associated genes at transcription levels

**FIGURE 3:**
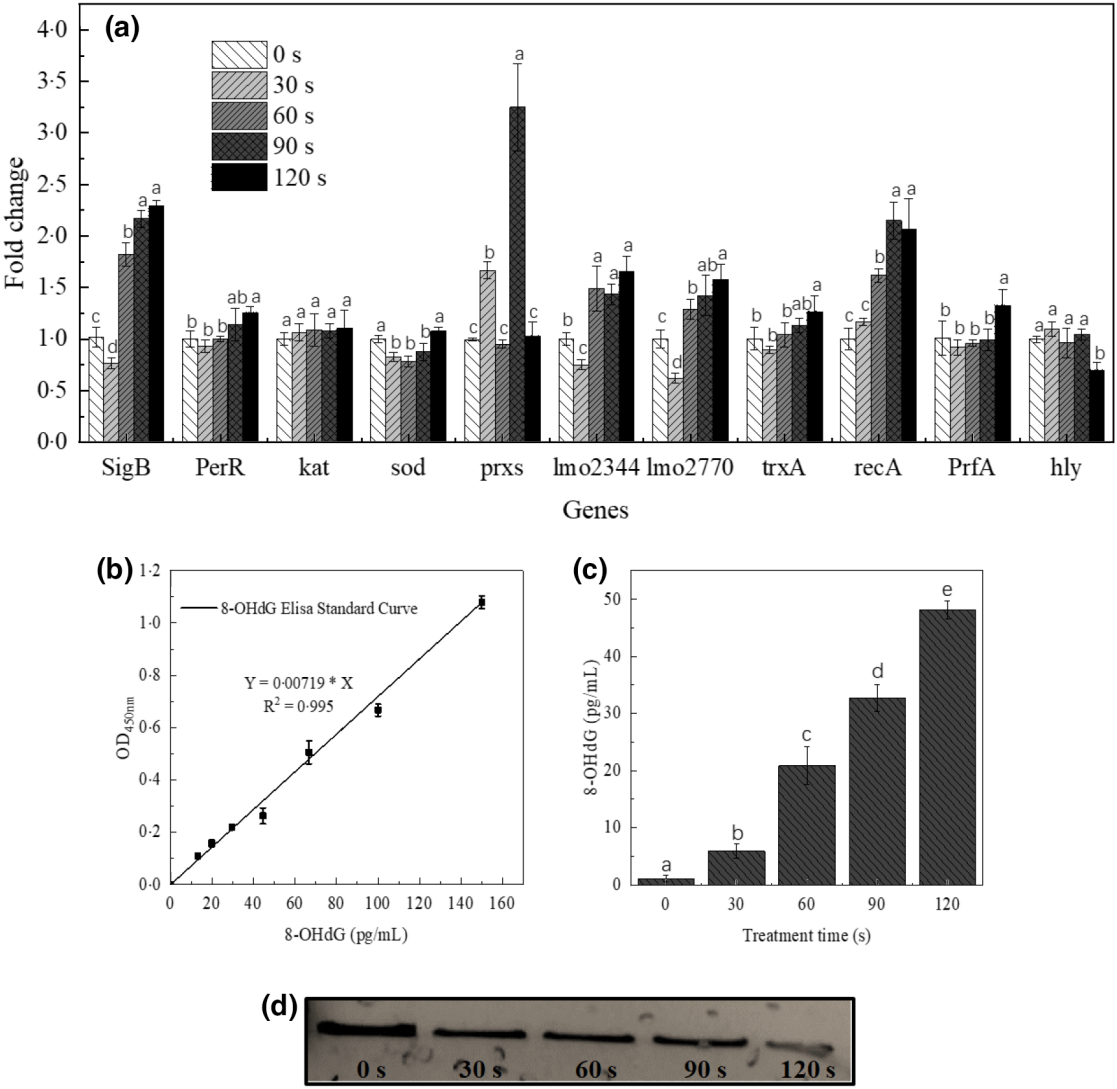
(a) Fold change of stress/virus‐associated genes relative expression in *L. monocytogenes* after exposure to plasma treatment; (b) the standard curve of 8‐OHdG with serially diluted solutions by ELISA; (c) the accumulation of 8‐OHdG in *L. monocytogenes* after plasma treatment for different times; (d) agarose gel electrophoresis (0.8%) of genomic DNA extracted from *L. monocytogenes* treated with DBD plasma for different times. The values given in the figure represent the mean ± SD of triplicate samples. Values in the same figure or gene with different letters were significantly different (*p* < 0.05)

Stress responses in *L. monocytogenes* have been extensively reported, but investigations pertaining to cellular responses to cold plasma‐mediated oxidative stress at transcription levels to explain the results in the current study are still limited. Therefore, one general stress‐regulator encoding gene (*sigB*), seven oxidation resistance genes (*perR*, *kat*, *sod*, *prxs*, *lmo2344*, *lmo2770* and *trxA*), one DNA repair gene (*recA*) and two virulence genes (*prfA*, and *hly*) were adopted for transcriptional analysis after plasma treatments for different times. The majority of these candidate reference genes were upregulated significantly (*p* < 0.05), with the exception of the virulence gene (*hly*) (Figure 3a).

The data indicated that the relative expression levels of general stress transcription factor *sigB* decreased initially and then increased steadily with prolonged treatment time (*p* < 0.01). Plasma treatment for 120 s showed elevated expression levels of *sigB* by 2.25‐fold as compared with untreated controls. More interestingly, mild oxidative stress from exposure time of 30 s induced downregulated transcription levels of *L. monocytogenes* in *sigB* and antioxidant‐related genes (*sod*, *lmo2344* and *lmo2770*) (*p* < 0.05), which might be due to the short‐term incompatibility of *L. monocytogenes* in mild oxidative stress. For antioxidative *kat* and *sod* genes in *L. monocytogenes*, only the *sod* gene was obviously upregulated after plasma exposure for 120 s (*p* > 0.05). Regarding *perR*, its transcription abundance also decreased initially (*p* > 0.05) and then increased steadily with prolonged plasma treatment time (*p* < 0.01).

In addition, plasma exposure for 90 s resulted in visible expression elevation of *recA* in *L. monocytogenes* (*p* < 0.01). Regarding the transcription levels of two virulence genes (*prfA*, *hly*), no significant fluctuation was observed in *prfA* within the first 90 s of plasma treatment (*p* > 0.05) until 120 s (*p* < 0.01). *PrfA* is a central virulence regulator of virulence gene transcription in *L. monocytogenes*, and its transcriptional activation is affected by the transcriptional levels of *sigB* (Pereira et al., 2018). However, a significant decrease in the expression of *hly* was observed with progressing in plasma treatment (*p* < 0.01), indicating a possible decline in LLO expression and the capacity of *L. monocytogenes* to cause infection.

### Genomic DNA lesions

To confirm that the elevated expression of *recA* in Figure 3a was due to the repair requirements of DNA lesions, the integrity and oxidative damages of genomic DNA were determined in the current study after plasma exposure. The accumulation of 8‐OHdG in *L. monocytogenes* during plasma treatment is shown in Figure 3c, indicating that the intracellular accumulation of 8‐OHdG exhibited a time‐dependent behaviour, and exposure time of 60 s was significant to elevate the content of 8‐OHdG to 20.83 ± 3.29 pg/ml (*p* < 0.01). With progressing exposure time from 0 s to 120 s, a 2.41‐fold elevation (48.13 ± 1.62 pg/ml) was observed, confirming the oxidative lesions of DNA. In addition, with prolonging plasma treatment time, the grey intensity of agarose gel was decreased along with the fragmentation of genomic DNA of *L. monocytogenes* (Figure 3d).

### Haemolytic activity

**FIGURE 4:**
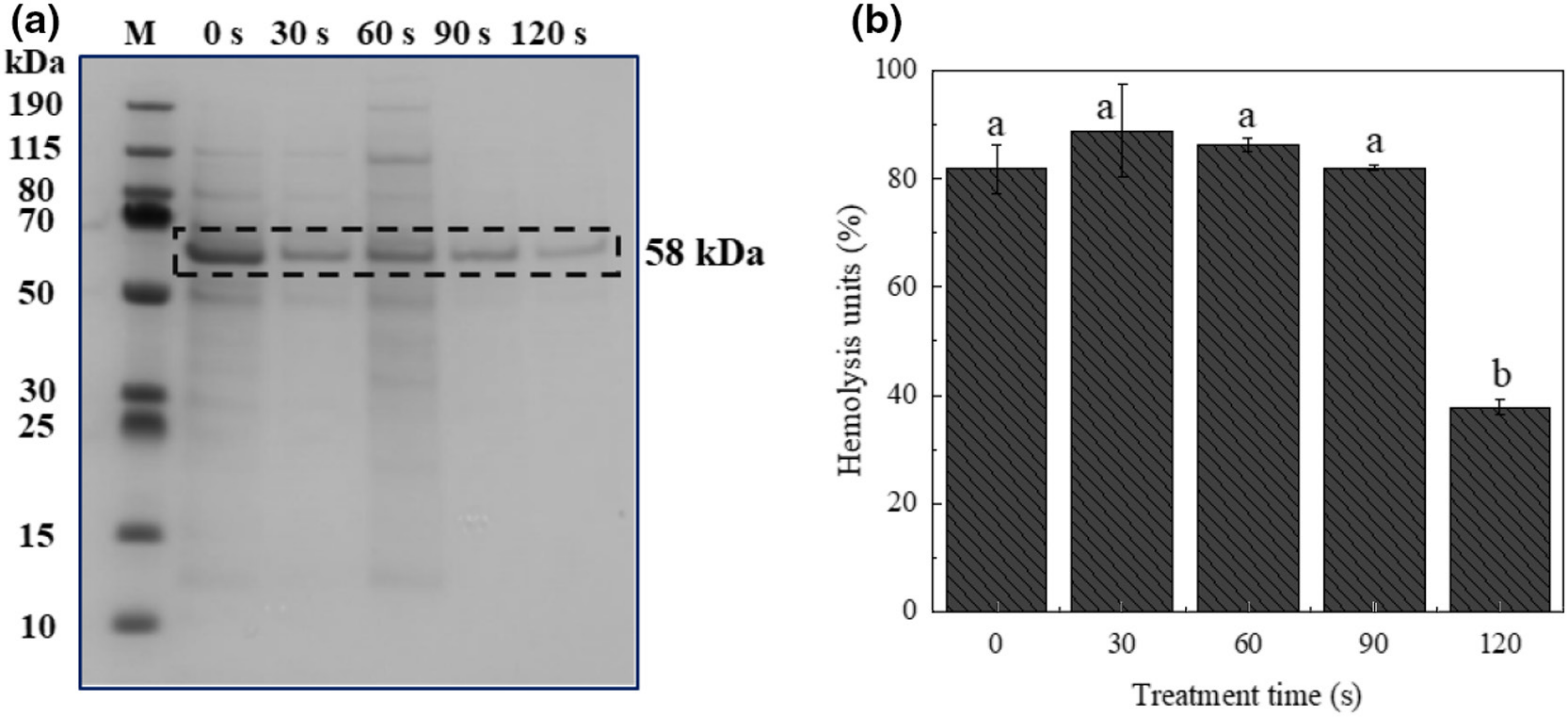
(a) Detection of the secretion of extracellular proteins in culture supernatants of *L. monocytogenes* after DBD plasma treatment for different times. Secreted proteins were separated in the 4–12% SDS‐PAGE gel and M referred to a marker as well as the band at 58 kDa corresponding to LLO; (b) haemolytic activity was performed with *L. monocytogenes* culture supernatants after plasma treatment for different times. The values given in the figure represent the mean ± SD of triplicate samples. Values in the same figure with different letters were significantly different (*p* < 0.05)

SDS‐PAGE assay was performed to evaluate the secretion of extracellular proteins of *L. monocytogenes* exposed to plasma treatment, and the haemolytic activities were detected by a listeriolysin assay. As shown in Figure 4a,b, compared with the control group, the SDS‐PAGE assay indicated that an outstanding difference was observed in the secretion of extracellular proteins from the supernatant of *L. monocytogenes* within the 120 s of plasma treatment. However, no significant decline but a slightly increased expression was observed in the intensity of the protein band located at 58 kDa within the first 90 s of plasma treatment, and the reduced intensity at 120 s indicated a possible secretion decline in the expression of virulence protein (Listeriolysin O). Meanwhile, the haemolytic activity of *L. monocytogenes* strains was reduced to 37.81 ± 1.53% when exposure was extended to 120 s (*p* < 0.01).

### Bacterial respiration

**FIGURE 5:**
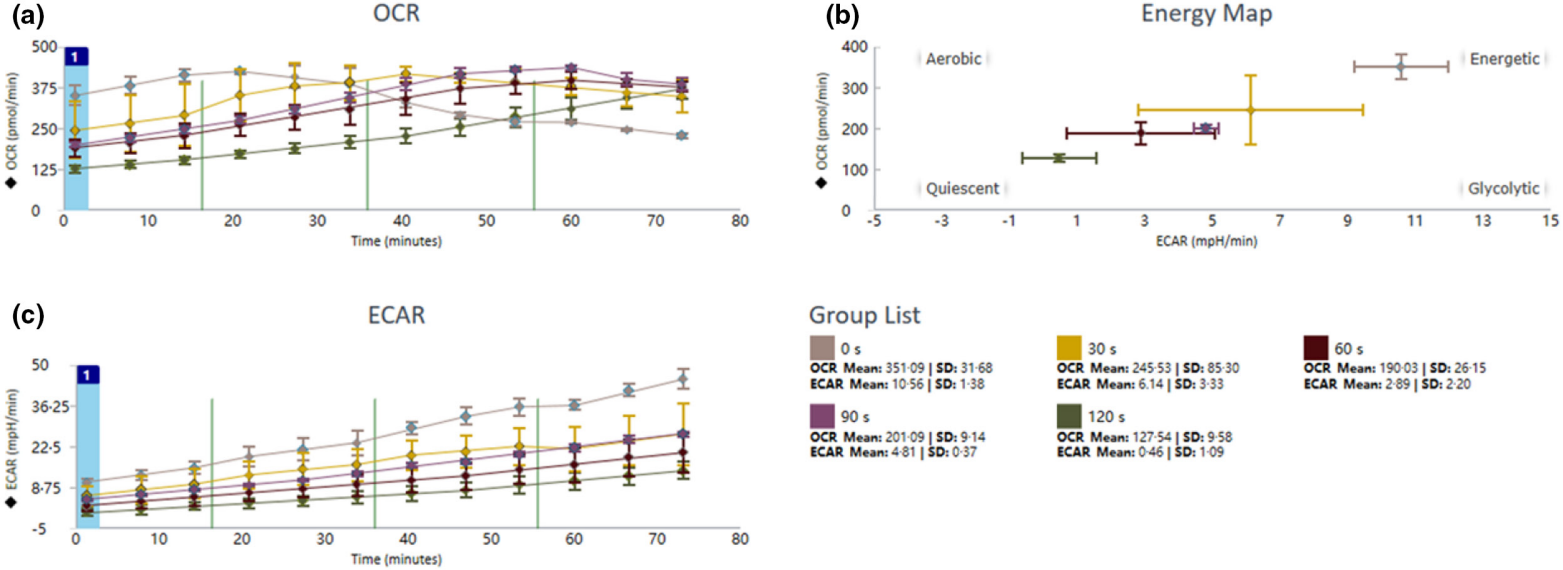
Real‐time changes in (a) oxygen consumption rate (OCR) and (b) extracellular acidification rate (ECAR) as well as (c) energy map in response to plasma exposure in *L. monocytogenes* as measured using a seahorse XFe extracellular flux Analyser

As shown in Figure 5, a Seahorse XFe Extracellular Flux Analyser was adopted to measure real‐time changes in OCR and ECAR as well as energy map in response to plasma exposure in *L. monocytogenes*. Immediately following plasma exposure, both the OCR and ECAR values and the OCR/ECAR ratios significantly dropped as plasma treatment time progressed to 120 s. Interestingly, during the post‐treatment storage for 75 min, there was a consistent instant elevation of OCR and ECAR values of *L. monocytogenes*, except for the OCR values of the control group (0 s). Meanwhile, even though the ECAR values of all plasma‐treated *L. monocytogenes* were still lower than the control, the OCR values of plasma‐treated groups were even higher than the untreated control group after a brief post‐storage of 75 min.

### Survival and growth of *L. monocytogenes* in raw milk

Figure 2c shows the post‐treatment viability of *L. monocytogenes* in raw milk at 4°C after plasma exposure. Generally, plasma exposure time had a significant impact on the recovery and growth speed of *L. monocytogenes* in raw milk. The exposure time of 60 s yielded a 0.82 ± 0.02 log reduction of viability with a 1.35 log increase over 7 days, demonstrating that a mild oxidative stress‐induced VBNC state had the potential to recover from the injury during resuscitation and became culturable in real food systems.

The growth of *L. monocytogenes* in raw milk was well controlled as the exposure time progressed to 90 s and a slight reduction was observed within 7 days. The exposure time of 120 s was more effective for significantly decreasing the population of *L. monocytogenes* as significant differences were found in the 120 s plasma‐treated group during recovery (*p* < 0.05). The further reduction in the level of *L. monocytogenes* in the raw milk during storage confirmed that this environment was not conducive for the growth of *L. monocytogenes* and recovery was not possible within 7 days.

## DISCUSSION

The results of the current study established DBD plasma as a significant source of ample exogenous RONS, that initiated the accumulation of main extracellular long‐lived species and intracellular oxidative stress (Han et al., 2019; Jiang et al., 2020). In fact, more than 75 species and 500 reactions occurred in the gas phase (Chen et al., 2020; Ekezie et al., 2017; Ekezie et al., 2018; Xu et al., 2017), but most of these highly reactive species in the gas phase were not detected by OES even though they had complicated reactions with the liquid system. Particle collisions that caused the quenching of O (^5^P) and O (^3^P) energy were responsible for the observed low optical transitions of the O atom (Walsh et al., 2010). Studies have suggested that H_2_O_2_, NO_3_^−^ and NO_2_^−^ are the main long‐lived species in plasma gas–liquid interaction (Xiang et al., 2018; Zhu et al., 2022), while ORP, H_2_O_2_ and NO_3_^−^ were the three predominant germicidal factors affecting the inactivation efficiency of plasma‐activated water (PAW) (Shen et al., 2016; Thirumdas et al., 2018). Among them, H_2_O_2_ is formed at the gas/liquid interface via two reactions (e^−^ + H_2_O → H· + OH· + e^−^; OH· + OH· → H_2_O_2_) (Lukes et al., 2014), which as a dominant antibacterial agent causes the oxidation of bacterial cell components, such as DNA, proteins and membrane lipids (Anjem & Imlay, 2012). Hence, plasma‐mediated extracellular exogenous RONS can trigger the surge of intracellular endogenous oxidative stress that penetrates directly through the membrane into cells. Thus, even though bacterial cells are equipped to confront exogenous RONS, the two‐pronged methods of combining endogenous and exogenous RONS from plasma exposure enhanced the oxidative damage to *L. monocytogenes* cells. Therefore, the DBD plasma could induce the oxidative environment and contribute to different inactivation efficiency.

The potential of virulence attenuation was observed in the assays of SDS‐PAGE and haemolytic activity determination as well as virulence gene expression at physiological and molecular levels. Usually, in the absence of the LLO‐encoding *hly* gene, *L. monocytogenes* is avirulent to murine models because *L. monocytogenes* cannot proliferate in mammalian cells (Pushkareva & Ermolaeva, 2010), which means that the downregulation of *hly* transcription levels in *L. monocytogenes* caused by plasma treatment is of interest to food safety. However, *hly* (the gene encoding LLO) was notably inhibited in the transcriptional levels in a time‐dependent manner (*p* < 0.01), while *L. monocytogenes* in this oxidative adaptation phase (plasma treatment less than 90 s) did not show a corresponding significant decrease in the haemolysis assay of LLO (*p* > 0.05). These results manifested that the downregulation of transcriptional levels did not imply the instant downregulation of the activities or expression of associated virulence proteins. An inconsistency between transcriptional levels and associated protein activity was also observed by Boura et al. (2016), who found that the wild type and ∆*sigB* mutant differed in catalase activities, but there was no difference observed in transcriptional levels of the *kat* gene.

At the same condition, our previous study indicated that plasma treatment for 90 s caused a certain degree of the inactivation of *L. monocytogenes* (Pan et al., 2020). However, no corresponding significant decline in haemolytic activity was noticed in the current study until plasma treatment time was extended to 120 s, and the haemolytic activity even slightly increased during the first 30 s plasma treatment (Figure 4b). Previous research demonstrated the potential of plasma treatment to attenuate the virulence of several foodborne pathogens, such as *Pseudomonas aeruginosa*, *Staphylococcus aureus*, *Escherichia coli* and *L. monocytogenes* (Flynn et al., 2016; Ziuzina et al., 2015). Non‐thermal cold plasma is capable of attenuating quorum sensing (QS)‐dependent virulence in microbes possibly due to induced degradation of acyl‐homoserine lactone (AHL) molecules by RONS, which enables QS‐dependent virulence attenuation in bacterial strains (Flynn et al., 2016). The results provided a new perspective for optimizing the operating parameters of plasma systems as well as assessing or promoting food safety rather than just from the aspect of inactivation efficiency. However, further investigation is still warranted to provide direct evidence to explain the mechanisms of the diminishment of the virulence of pathogenic bacteria and to determine the treatment needed.

8‐OHdG as an oxidized form of guanine is a ubiquitous marker and the by‐product of oxidative stress, and its amount indicates the extent of DNA lesions (Barzilai & Yamamoto, 2004). Analogous formation of 8‐OHdG by plasma‐mediated oxygen radicals was observed by Yost and Joshi (2015) and Joshi et al. (2011). Yost and Joshi (2015) found that non‐thermal plasma treatment for 1 min caused a significant difference between the level of 8‐OHdG in wildtype (14 pg/ml) and in Δ*sodA* and Δ*katE* mutants (38 pg/ml and 87 pg/ml, respectively) of *Escherichia coli*. Besides, our previous study had reported the absence of rupture to *L. monocytogenes* after cold plasma exposure for 2 min and taken together with the oxidative lesions of genomic DNA from the current study, it could be inferred that the inactivation mechanism of gram‐positive bacteria is mainly due to the intracellular macromolecular damage (Han et al., 2015; Pan et al., 2020).

Even though obvious oxidative damage of DNA and accumulation of 8‐OHdG were observed, a quick recovery of *L. monocytogenes* in milk was also noticed after exposure to the plasma‐mediated mild oxidative environment. This may be attributed to the upregulation of DNA repair gene (*recA*) and oxidation resistance genes (e.g. *sigB*, *perR* and *sod*). Maintaining the integrity of the arrested replication fork and restoring the genomic template by upregulating more than 40 associated genes and then promoting the recovery of progressive replication, such as activation translation DNA synthesis polymerases and DNA repair mechanisms, have been suggested as the major functions of *recA* (Courcelle & Hanawalt, 2003). These functions have also been substantiated by van der Veen et al. (2010), who showed that ∆*recA* mutant strains were reduced by 3 logs after 1 h exposure to 60 mM H_2_O_2_ and the mutant strains were more sensitive to H_2_O_2_‐induced oxidative stress than the wild‐type strains. Therefore, a hypothesis could be proposed from the elevated *recA* that *recA*‐controlled functions of *L. monocytogenes* were activated after exposure to plasma‐mediated oxidative stress. The *recA*‐controlled functions were likely to facilitate the repair of DNA lesions and were also involved in oxidative stress survival. In addition, despite the well‐established function of *sigB* in regulating the expression levels of more than 150 related genes, which contributes to multiple‐stress resistance of *L. monocytogenes* to adverse environmental conditions such as oxidative stress, osmotic stress, heat shock, acid stress (Boura et al., 2016), the result in the current work indicated the crucial role of *sigB* in *L. monocytogenes* as an oxidative stress regulator in preventing the damage caused by reactive oxygen species such as free radicals and peroxides. Oliver et al. (2010) also mentioned that oxidative stress protection was conferred by *sigB* in *L. monocytogenes* since ∆*sigB* mutant strains were less resistant to oxidative stress as compared with the wild‐type strains. Regarding elevated *perR*, it plays an essential role both in routine detoxification of endogenously produced peroxides and exogenous ROS stress. Antioxidative *kat* genes in *L. monocytogenes* have a synergistic effect with the dominant *sod* gene, both of which show implications in the protection against plasma‐mediated ROS. Typically, the superoxide dismutase encoded by the *sod* gene acts by dismutating the superoxide radical anion O_2_^•^‐ to H_2_O_2_, which is then converted into H_2_O by catalase encoded by the *kat* gene (Pieta et al., 2017). And the elevated expression of *sod* in this study was in agreement with other studies related to the oxidative stress response (Yost & Joshi, 2015). Therefore, as described above, oxidative stress‐induced upregulation of the signalling of antioxidant‐related genes in *L. monocytogenes* could contribute to the resistance of microorganisms in oxidative conditions, which are suitable for their survival.

Interestingly, a 2.75‐fold reduction of OCR was observed in *L. monocytogenes* after plasma exposure for 120 s, indicating uncoupling of respiration from ATP production and a compensatory drop in respiration (Lobritz et al., 2015) (Figure 5a). Measurement of the ECAR of this strain further confirmed a substantially lower rate of acid secretion after plasma treatment (Figure 5b), which indicated the suppression of glycolysis. In addition, a dynamic shift in OCR/ECAR ratio was observed after plasma exposure at different times (Figure 5c), which implied that plasma treatment resulted in *L. monocytogenes* being driven to a lower energy state of metabolic activity. Similar results were reported by Liao et al. (2021), showing that cold plasma exposure triggered and forced bacterial cells to change their energy allocation and suppress other energy‐dependent physiological activities (e.g. cell wall biosynthesis, metabolism) to achieve a ‘low‐energy‐consumption’ survival pattern of viable but non‐culturable (VBNC) state. Studies have shown that sublethal injury or VBNC state is universally accepted and confirmed as the phenomenon during plasma treatment (Millan‐Sango et al., 2015; Pan et al., 2019b). More interestingly, compared with the control group, there were instantly ever‐elevating and even higher OCR values in the plasma‐treated *L. monocytogenes* during a brief post‐storage for 75 min, which indicated that an instantly intense over‐oxidative response was triggered. However, the persistence of the over‐oxidative response was not clear. Therefore, the survival assay for plasma‐treated bacteria especially in real food systems is necessary to evaluate the potential risk during post‐treatment storage. Conventional viable counts overestimated the efficiency of plasma inactivation (Dolezalova & Lukes, 2015), and the survival assay in raw milk suggested the resuscitation potential and safety risk of mild plasma inactivated *L. monocytogenes*. A similar recovery assay of *L. monocytogenes* was reported by Alessandria et al. (2019) after cold atmospheric pressure plasma treatments for 10 min. Even though conventional viable counts indicated the inactivation efficiency of plasma treatment, the quantitative PCR method suggested the presence of injured cells or their VBNC state and *L. monocytogenes* could recover quickly at 37°C in brain heart infusion broth within 24 h. The fact that cells could recover quickly after the treatment suggested that *L. monocytogenes* overcame the injury mediated by cold plasma during the post‐storage, which was reversible and may contribute to further microbial proliferation, increasing the risk of human exposure to the contaminated water or foods. Plasma treatment was effective for reducing the level of *L. monocytogenes* but conventional methods commonly overestimated the decontamination efficiency of the method, and plasma‐treated foodborne pathogens were able to resuscitate, revealing the potential risks to the health of consumers. Therefore, suitable plasma time was needed to control the VBNC state and more molecular methods are necessary to confirm the total inactivation of foodborne pathogens. In the current study, it was recommended that plasma exposure time longer than 120 s was more suitable for attenuating virulence and avoiding the recovery possibility in raw milk within 7 days. In future, more attention should be paid to plasma dose control and plasma‐mediated possible VBNC or injured state. Additional studies are also needed to evaluate the effect of food that cold plasma treatment might have, along with the genetic toxicity of plasma‐treated *L. monocytogenes* and the attenuation mechanism.

## CONFLICT OF INTEREST

Authors declare that he/she has no conflict of interest.

## Data Availability

Data are available within the article or its supplementary materials.
